# Supplementary material for: The interactome of intact mitochondria by cross-linking mass spectrometry provides evidence for coexisting respiratory supercomplexes
Source: Mol Cell Proteomics. 2017 Dec 8;17(2):216–32. doi: 10.1074/mcp.RA117.000470 (PMC5795388; doi:10.1074/mcp.RA117.000470)
Supplement: Supplemental Data [file supp_RA117.000470_133922_0_supp_23978_fzffwf.zip › spectra_annotation/mito_DR_spectra_annotation/464-1-10-1-4-1.pdf]

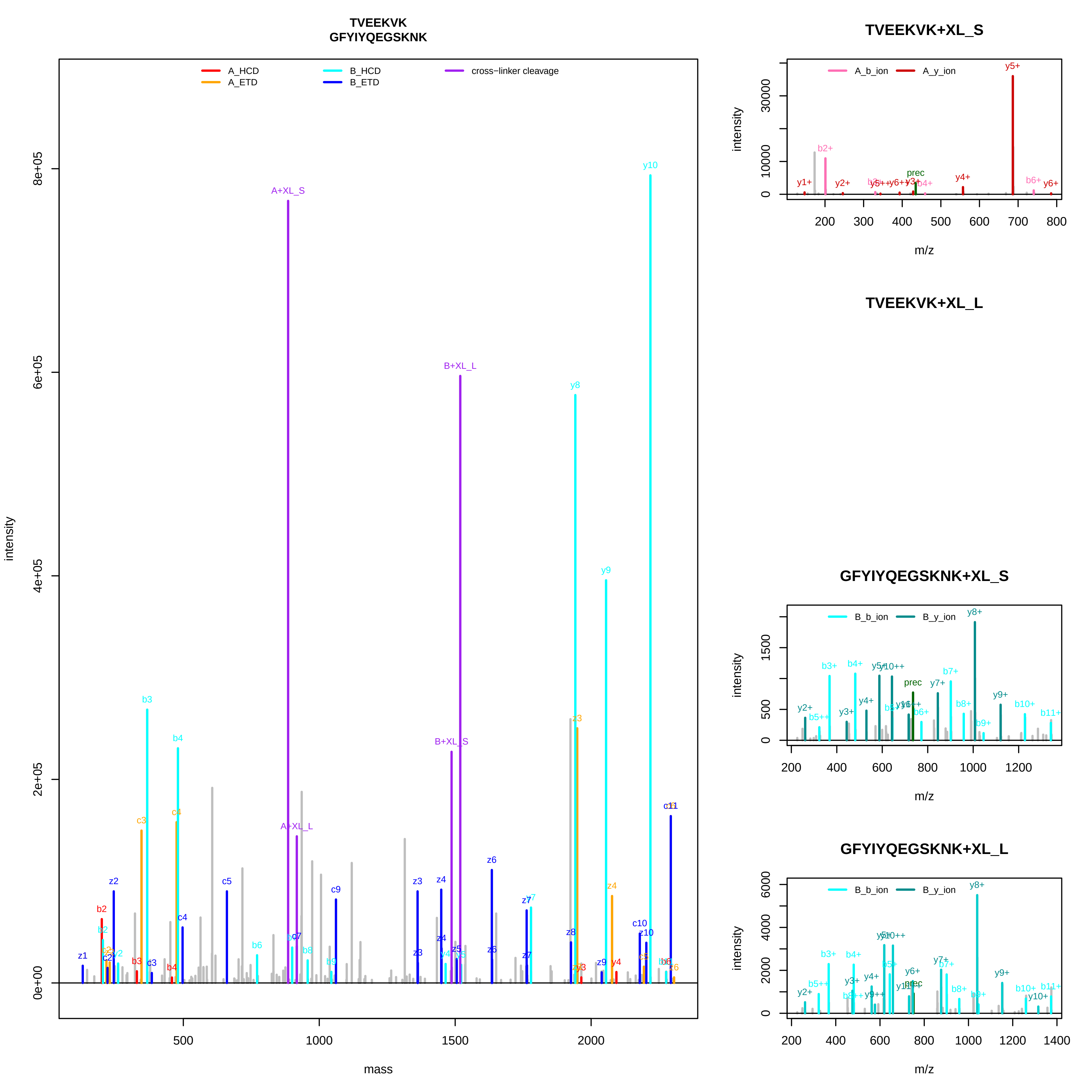
TVEEKVK
GFYIYQEGSKNK
A_HCD
A_ETD
B_HCD
B_ETD
cross−linker cleavage
intensity
mass
0e+00
2e+05
4e+05
6e+05
8e+05
500
1000
1500
2000
A+XL_S
B+XL_L
B+XL_S
A+XL_L
y10
y8
y9
b3
b4
c3
c4
z3
z4
c11
z6
z2
c5
c9
z7
b2
TVEEKVK+XL_S
A_b_ion
A_y_ion
y5+
b2+
prec
y4+
y1+
y2+
b6+
y6+
m/z
TVEEKVK+XL_L
GFYIYQEGSKNK+XL_S
B_b_ion
B_y_ion
y8+
b3+
b4+
b7+
y7+
y9+
b10+
b11+
GFYIYQEGSKNK+XL_L
